# Addressing Food Insecurity and Climate Change in Malaysia: Current Evidence and Ways Forward

**DOI:** 10.21315/mjms2022.29.6.1

**Published:** 2022-12-22

**Authors:** Jemilah Mahmood, Nadia N Rajaram, Renzo R Guinto

**Affiliations:** Sunway Centre for Planetary Health, Sunway University, Selangor, Malaysia

## The Impact of COVID-19 and Climate Change

Access to sufficient, nutritious food is an urgent, mounting global problem that has been exacerbated by the COVID-19 pandemic. In 2020, up to 30% of the global population faced food insecurity, a 4% increase from the preceding year, with great variation across regions (1). The highest levels of food insecurity were reported in the African continent, where more than half the population (59%) reported poor access to food (1). The largest impact of the pandemic, however, was observed in Latin America and the Caribbean, where the pandemic led to an almost 10% increase in food insecurity in just over a year, resulting in 41% of the population living with food insecurity (1). In Asia, the prevalence of food insecurity increased by 3% to 26% in 2020 (1). The pandemic highlighted how vulnerable current food systems are, especially in emerging economies that rely on large-scale agriculture and international food trade. Without intervention, the global food-insecure population is expected to rise by another 10% by 2050 (2), or more if another global catastrophe strikes.

Food insecurity is both a product and a driver of climate change, the effects of which are most felt in low- and middle-income countries where food systems are already at risk (3). The increasing severity of climate change, such as high surface temperatures, rising sea levels, and more frequent extreme weather events, disrupt food systems and increase food spoilage, leading to decreased food supply (4). Additionally, soil erosion and increased carbon dioxide levels lead to changes in the nutrient content in food, thereby affecting the quality of food produced (5). Concurrently, the production of animals and animal products for human consumption is responsible for up to one third of global greenhouse gas emissions, a major contributor to climate change (6). This vicious cycle of increasing climate change and growing food insecurity leads to more hunger and higher levels of malnutrition, and increases the number of people facing avoidable death and disability (7).

### Hunger, Undernutrition and Noncommunicable Disease in Malaysia

Malaysia is no exception to the growing threat of rising food insecurity. In the 2022 Global Hunger Index, Malaysia scored moderately for hunger, with a small increase in the hunger index from 10.9 in 2014 to 12.5 in 2022 (8), likely corresponding to a rise in food insecurity because of the pandemic. Data from the Malaysian Adult Nutrition Survey 2014 showed that up to 25% of the population faced insufficient food quantity and variety at that time, with a higher prevalence observed among low-income households (33%–39%) (9). In this survey, up to 22% of respondents reported ever having to reduce their meal size or skip a meal due to financial constraints in the past year, whilst 21% of respondents reported ever having to feed their children with less food variety (9). This prevalence is likely higher in 2022, as evidenced by the increase in stunting and wasting among children in Malaysia, by 4% and 2% respectively, since 2014 (8). If Malaysia is unable to ensure equitable access to affordable and nutritious food, it is anticipated that the food security gap will reach 40% over the next 40 years (10).

Equally worrying, the trend in food consumption in Malaysia has been changing over the past several decades to a pattern that is problematic for population health and the environment. The demand for animal-based proteins increased by 59% between 1980 and 2014, and has surpassed the demand for plant-based sources of protein (11). In Malaysia, up to 90% of red meat consumption is not locally sourced and the industry relies on import to meet current demand, significantly increasing the carbon cost of getting meat to table (12). Another alarming food trend is the increase in demand for sugar and sweeteners, where supply of these items increased by 24% between 1980 and 2014. Furthermore, the recent National Health and Morbidity Survey (NHMS) indicated that up to 95% of adults do not consume adequate amounts of fruits and vegetables in their diet (13). Limited availability and affordability of sufficient, nutritious food may be leading to unhealthy eating habits, as cheaper, more enticing, ultra-processed foods become more accessible.

The increasingly carbon-intensive food systems are the primary drivers of the non-communicable disease epidemic (14), including here in Malaysia. The 2019 NHMS reported that half of the population were already overweight or obese and without immediate intervention, this figure is projected to continue rising (13). Up to 30% of Malaysian adults suffer from diabetes and/or hypertension, with half of them unaware, undiagnosed and untreated (13). Cardiovascular disease, cancer and diabetes accounted for over 2 million disability-adjusted life years lost and an estimated productivity loss of up to RM13 billion in 2017 (15). Hence, the rehabilitation of food systems in Malaysia represents an opportunity to tackle food insecurity and the rising burden of non-communicable diseases in Malaysia, in addition to addressing climate change in the country.

### Climate Change and Food Insecurity in Malaysia

Evidence of climate change in Malaysia, as well as implications on the agriculture industry, has become glaringly apparent in recent years. Extreme weather events have become more common, such as the unprecedented major flooding in 2014 (Kedah) and 2021 (Selangor). The 2014 floods resulted in RM299 million worth of losses due to damage to crops, natural resources and infrastructure (16). In the country’s primary granary, recent air temperature has been recorded as high as 40 °C during the dry season, compared to an average of 31 °C–34 °C, leading to shortages in water reservoirs for irrigation and poor conditions for growing rice (17, 18). Extreme weather events are expected to reduce rice yield by up to 31% within the next decade (19) and reduce the country’s capacity to maintain rice self-sufficiency levels above the minimum national requirement for self-sufficiency of 65% (17).

Unsurprisingly, the impact of climate change and food insecurity are most keenly felt by those who are poor, already on the brink of food insecurity and most often working in the agriculture and food production industries. In Malaysia, the most vulnerable populations reside in Kelantan and Sabah, the two states whose economies rely heavily on agricultural activities (20). With climate change-induced disruption to agriculture, workers in the industry face reduced income, further thrusting them into poverty and food insecurity (19). Furthermore, this group is most vulnerable to sudden shocks to the food production systems, as observed during the COVID-19 pandemic where people who were already facing food insecurity faced even deeper levels of that insecurity (21).

### Curb, Adapt, Monitor and Advocate

What can Malaysia do to better meet the changing needs of its people and the planet? Firstly, there is an urgent need to **curb** further environmental change in Malaysia. This includes introducing major changes to our food systems that focus on a shift to plant-based diets, reduction in food waste and evidence-based improvements to food production practices (22). Adopting dietary patterns that are healthier and encourage sustainability of our environment and resources, such as a Planetary Health Diet (PHD), can reduce the demand for animal sources of protein as well as sweetened and ultra-processed foods (23). Successful adoption of the PHD in Malaysia is expected to reduce premature mortality by up to 20% and reduce environmental resource demands by up to 30% (24).

Secondly, the agricultural industry must **adapt** to the effects of climate change on food production systems, such as by increasing crop diversity to ensure greater resilience to extreme weather events. It was projected that adaptation of agricultural practices in Malaysia will only cost a small percentage of the Malaysia’s GDP (0.00055%), especially if appropriate interventions are employed at an early stage (10). Thirdly, and in parallel, Malaysia must prioritise improvements in data collection and analytics to **monitor** progress—or lack thereof—made towards environmental change. Lastly, we must recognise the role of healthcare professionals to **advocate** for action in this unique nexus of food, environment and health (22).

Malaysia’s food systems are critically vulnerable to environmental change and may not be able to withstand future catastrophic events, the brunt of which will be borne by the poorest sectors of the population. Evidence-based reforms to food systems are urgently needed to allow the country to adapt, mitigate and repair the negative effects of climate change, leading to a healthier population and planet.

## References

[1] Food and Agriculture Organization of the United Nations (2021). The state of food security and nutrition in the world 2021 [Internet].

[2] Nelson G, Bogard J, Lividini K, Arsenault J, Riley M, Sulser TB (2018). Income growth and climate change effects on global nutrition security to mid-century. Nat Sustain.

[3] Green R, Scheelbeek P, Bentham J, Cuevas S, Smith P, Dangour AD (2022). Growing health: global linkages between patterns of food supply, sustainability, and vulnerability to climate change. Lancet Planet Health.

[4] Benton TG, Castro De La Mata G, Fanzo J, Guinto R, Hendriks SL, Montgomery H (2022). Food security and health in a changing environment: recognizing and mitigating risks.

[5] Zhu C, Kobayashi K, Loladze I, Zhu J, Jiang Q, Xu X (2018). Carbon dioxide (CO_2_) levels this century will alter the protein, micronutrients, and vitamin content of rice grains with potential health consequences for the poorest rice-dependent countries. Sci Adv.

[6] Xu X, Sharma P, Shu S, Lin TS, Ciais P, Tubiello FN (2021). Global greenhouse gas emissions from animal-based foods are twice those of plant-based foods. Nature Food.

[7] Men F, Gundersen C, Urquia ML, Tarasuk V (2020). Association between household food insecurity and mortality in Canada: a population-based retrospective cohort study. CMAJ.

[8] Global Hunger Index (GHI) (2022). Global Hunger Index 2022.

[9] Institute for Public Health (2014). National Health and Morbidity Survey 2014: Malaysian adult nutrition survey. Volume II: survey findings.

[10] Ahmed F, Al-Amin AQ, Mohamad ZF, Chenayah S (2016). Agriculture and food security challenge of climate change: a dynamic analysis for policy selection. Sci Agric.

[11] vonGoh E, Azam-Ali S, McCullough F, Roy Mitra S (2020). The nutrition transition in Malaysia; key drivers and recommendations for improved health outcomes. BMC Nutr.

[12] Zayadi RA (2021). Current outlook of livestock industry in Malaysia and ways towards sustainability. J-SuNR.

[13] Ministry of Health Malaysia (2020). National Health and Morbidity Survey 2019Technical report-volume 1 NCDs: risk factors and other health problems.

[14] Lane MM, Davis JA, Beattie S, Gómez-Donoso C, Loughman A, O’Neil A (2021). Ultraprocessed food and chronic noncommunicable diseases: a systematic review and meta-analysis of 43 observational studies. Obes Rev.

[15] Ministry of Health Malaysia (2020). The impact of non-communicable diseases and their risk factors on Malaysia’s gross domestic product.

[16] (2015). Agriculture sector suffers RM299mil loss due to floods [Internet]. The Star.

[17] Jusop S (2022). Science matters: climate change affects food security [Internet]. The Edge Malaysia.

[18] Perak MB (2022). Drop in Bukit Merah Dam water level affecting paddy farmers, industrial sector [Internet]. malaymail.com.

[19] Vaghefi N, Shamsudin MN, Radam A, Rahim KA (2015). Impact of climate change on food security in Malaysia: economic and policy adjustments for rice industry. J Integr Environ Sci.

[20] Yusuf AA, Francisco H (2009). Climate change vulnerability mapping for Southeast Asia.

[21] Béné C (2020). Resilience of local food systems and links to food security: a review of some important concepts in the context of COVID-19 and other shocks. Food Secur.

[22] Guinto RR, Baluyot CJ, Gan CCR, Ghosh U, Daniel M, Mahadzir A (2022). Health sector solutions for promoting sustainable and nutritious diets. BMJ.

[23] Willett W, Rockström J, Loken B, Springmann M, Lang T, Vermeulen S (2019). Food in the Anthropocene: the EAT–Lancet Commission on healthy diets from sustainable food systems. Lancet.

[24] Springmann M, Spajic L, Clark MA, Poore J, Herforth A, Webb P (2020). The healthiness and sustainability of national and global food based dietary guidelines: modelling study. BMJ.

